# Antibiotic prescription behaviours in Lao People's Democratic Republic: a knowledge, attitude and practice survey

**DOI:** 10.2471/BLT.14.142844

**Published:** 2015-03-03

**Authors:** Fabrice Quet, Erika Vlieghe, Caroline Leyer, Yves Buisson, Paul N Newton, Philaysak Naphayvong, Valy Keoluangkhot, Monique Chomarat, Christophe Longuet, Nicolas Steenkeste, Jan Jacobs

**Affiliations:** a UMR 1094 (Université de Limoges Inserm/CHU de Limoges), Limoges, France.; b Institute of Tropical Medicine, Nationalestraat 155, 2000 Antwerp, Belgium.; c Fondation Mérieux, Lyon, France.; d Institut de la Francophonie pour la Médecine Tropicale (IFMT), Vientiane, Lao People's Democratic Republic.; e Lao-Oxford-Mahosot Hospital-Wellcome Trust Research Unit, Vientiane, Lao People's Democratic Republic.; f Laboratoire de Microbiologie Centre Hospitalier, Lyon-Sud Pierre-Bénite, France.

## Abstract

**Objective:**

To assess the antibiotic prescribing practices of doctors working in the Lao People's Democratic Republic and their knowledge of local antibiotic resistance patterns.

**Methods:**

Doctors attending morning meetings in 25 public hospitals in four provinces were asked to complete a knowledge, attitude and practice survey. The questionnaire contained 43 multiple choice questions that the doctor answered at the time of the meeting.

**Findings:**

The response rate was 83.4% (386/463). Two hundred and seventy doctors (59.8%) declared that they had insufficient information about antibiotics. Only 14.0% (54/386) recognized the possibility of cephalosporin cross-resistance in methicillin-resistant *Staphylococcus aureus*. Most participants had no information about local antibiotic resistance for *Salmonella* Typhi (211/385, 54.8%) and hospital-acquired pneumonia (253/384, 65.9%). Unnecessary antibiotic prescriptions were considered as harmless by 115 participants and 148 considered locally-available generic antibiotics to be of poor quality. Nearly three-quarters (280/386) of participants agreed that it was difficult to select the correct antibiotics. Most participants (373/386) welcomed educational programmes on antibiotic prescribing and 65.0% (249/383) preferred local over international antibiotic guidelines.

**Conclusion:**

Doctors in the Lao People's Democratic Republic seem to favour antibiotic prescribing interventions. Health authorities should consider a capacity building programme that incorporates antibiotic prescribing and hospital infection control.

## ملخص

الغرض ****تقييم ممارسات الأطباء العاملين في جمهورية لاو الديمقراطية الشعبية في وصف المضادات الحيوية ومعرفتهم بالأنماط المحلية لمقاومة المضادات الحيوية.

الطريقة ****طُلب من الأطباء الذين حضروا الاجتماعات الصباحية في 25 مستشفى عمومي في أربع مقاطعات المشاركة في مسح عن المعرفة والاتجاه والممارسة. وتضمن المسح 43 سؤالاً متعدد الخيارات أجاب عنها الطبيب أثناء الاجتماع.

النتائج ****كان معدل الاستجابة 83.4 % (386/463). وصرح مائتان وسبعون طبيباً (59.8 %) أنهم لا يمتلكون معلومات كافية عن المضادات الحيوية. وعرف 14.0 % فقط (54/386) إمكانية المقاومة المشتركة للسيفالوسبورين في البكتيريا العنقودية الذهبية المقاومة للميثيسيلين. ولم يكن لدى معظم المشاركين معلومات عن مقاومة المضادات الحيوية المحلية لبكتيريا السالمونيلا التيفية (211/385، 54.8 %) والالتهاب الرئوي الذي يحدث في المستشفيات (253/384، 65.9 %). واعتبر 115 مشاركاً وصفات المضادات الحيوية غير الضرورية آمنة واعتبر 148 مشاركاً المضادات الحيوية الجنيسة المتوفرة محلياً ذات جودة ضعيفة. واتفق ثلاثة أرباع المشاركين تقريباً (280/386) أنه كان من الصعب اختيار المضادات الحيوية المناسبة. ورحب معظم المشاركين (373/386) بالبرامج التثقيفية المعنية بوصف المضادات الحيوية وفضل 65.0 % (249/383) الدلائل الإرشادية المحلية للمضادات الحيوية عن الدلائل الإرشادية الدولية للمضادات الحيوية.

الاستنتاج ****يبدو أن الأطباء في جمهورية لاو الديمقراطية الشعبية يؤيدون تدخلات وصف المضادات الحيوية. وينبغي أن تفكر السلطات الصحية في برنامج لبناء القدرات يدمج بين وصف المضادات الحيوية ومكافحة العدوى بالمستشفيات.

## 摘要

**目的:**

评估老挝人民民主共和国医生的抗生素处方实务及其对当地抗生素耐药性模式的知识。

**方法:**

要求四个省25所公立医院参加晨会的医生完成知识、态度和实务调查。问卷包含43个多项选择题,医生在会议时间内完成回答。

**结果:**

响应率为83.4%(386/463)。其中270名医生(59.8%)称其了解足够的抗生素信息。只有14.0%医生(54/386)认识到在耐甲氧西林金黄色酿脓葡萄球菌中可能存在头孢菌素交叉耐药。大多数参与者对伤寒杆菌(211/385,54.8%)和院内肺炎(253/384,65.9%)的当地抗生素耐药性一无所知。115名参与者认为非必要的抗生素处方无害,148名认为当地可获得的一般抗生素质量较差。近四分之三(280/386)的参与者认为很难选择适合的抗生素。大多数参与者(373/386)对抗生素处方教育计划表示欢迎,65.0%(249/383)首选地方而非国际抗生素的指导方针。

**结论:**

老挝的医生似乎支持抗生素处方干预措施。卫生部门应该考虑推行包含抗生素处方和医院感染控制的能力建设计划。

## Résumé

**Objectif:**

Évaluer les pratiques de prescription d'antibiotiques des médecins travaillant en République démocratique populaire lao et leur connaissance des profils locaux de résistance aux antibiotiques.

**Méthodes:**

Les médecins participant aux réunions matinales dans 25 hôpitaux publiques dans 4 provinces ont été invités à répondre à une enquête sur leurs connaissances, leur attitude et leur pratique. Le questionnaire contenait 43 questions à choix multiples auxquelles les médecins répondaient au moment de la réunion.

**Résultats:**

Le taux de réponse était de 83,4% (386/463). Deux cent soixante-dix médecins (59,8%) ont déclaré ne pas avoir suffisamment d'informations sur les antibiotiques. Seuls 14,0% des médecins (54/386) ont admis la possibilité d'une résistance croisée pour la céphalosporine chez la bactérie *Staphylococcus aureus* résistante à la méthicilline. La plupart des participants n'avaient aucune information sur la résistance locale aux antibiotiques pour la bactérie *Salmonella* *Typhi* (211/385, 54,8%) et la pneumonie nocosomiale (253/384, 65,9%). Les prescriptions inutiles d'antibiotiques étaient considérées comme inoffensives par 115 participants et 148 participants considéraient que les antibiotiques génériques disponibles localement étaient de mauvaise qualité. Près de trois-tiers des participants (280/386) convenaient qu'il était difficile de sélectionner les antibiotiques appropriés. La plupart des participants (373/386) ont bien accueilli les programmes de formation relatifs à la prescription des antibiotiques et 65,0% (249/383) préféraient recevoir des recommandations locales pour les antibiotiques plutôt que des recommandations internationales.

**Conclusion:**

Les médecins en République démocratique populaire lao semblaient soutenir les interventions en matière de prescription d'antibiotiques. Les autorités de santé devraient envisager l'établissement d'un programme de renforcement des capacités qui intègre la prescription des antibiotiques et le contrôle des infections à l'hôpital.

## Резюме

**Цель:**

Оценить порядок назначения антибиотиков, которого придерживаются врачи, работающие в Лаосской Народно-Демократической Республике, а также осведомленность этих врачей о резистентности к антибиотикам местного действия.

**Методы:**

Присутствовавших на утренних совещаниях врачей в 25 государственных больницах четырех провинций попросили принять участие в исследовании осведомленности, отношения и практики. Им было предложено ответить на 43 вопроса с несколькими вариантами ответов во время совещания.

**Результаты:**

Частота ответов составила 83,4% (386/463). Двести семьдесят докторов (59,8%) заявили, что у них отсутствует достаточная информация об антибиотиках. Только 14,0% (54/386) принимает во внимание вероятность перекрестной резистентности к цефалоспорину при лечении метициллин-резистентного *золотистого стафилококка*. У большинства участников отсутствовала информация о резистентности к антибиотикам местного действия для лечения *сальмонеллы тифи* (211/385, 54,8%) и внутрибольничной пневмонии (253/384, 65,9%). Назначение антибиотиков без необходимости было расценено как вредное 115 участниками; 148 участников охарактеризовали непатентованные антибиотики, имеющиеся в местном доступе, как низкокачественные. Примерно три четверти (280/386) участников подтвердили существование трудностей при подборе правильных антибиотиков. Большинство участников (373/386) положительно относится к образовательным программам по назначению антибиотиков; 65,0% (249/383) предпочитает местные руководства по антибиотикам международным.

**Вывод:**

Можно заключить, что врачи в Лаосской Народно-Демократической Республике относятся к вмешательствам с назначением антибиотиков положительно. Органы здравоохранения должны рассмотреть возможность разработки программы по развитию и укреплению потенциала, включающей назначение антибиотиков и контроль за внутрибольничными инфекциями.

## Resumen

**Objetivo:**

Evaluar las prácticas de prescripción de antibióticos de los médicos que prestan servicio en la República Democrática Popular Lao y sus conocimientos de los patrones de resistencia a antibióticos locales.

**Métodos:**

Se solicitó a los médicos que asistían a reuniones por la mañana en 25 hospitales públicos de cuatro provincias que completaran una encuesta sobre conocimientos, actitudes y prácticas. El cuestionario contenía 43 preguntas de opción múltiple que los médicos contestaron en la reunión.

**Resultados:**

La tasa de respuesta fue del 83,4 % (386/463). 270 médicos (59,8 %) declararon que no disponían de información suficiente acerca de los antibióticos. Solo el 14,0 % (54/386) reconoció la posibilidad de resistencia cruzada con cefalosporina del *Staphylococcus aureus* resistente a la meticilina. La mayoría de los participantes no tenía información sobre la resistencia antibiótica local para la *Salmonela typhi* (211/385, 54,8 %) y la neumonía adquirida en el hospital (253/384, 65,9 %). 115 participantes consideraron inofensivas las prescripciones innecesarias de antibióticos y 148 consideraron que los antibióticos genéricos disponibles localmente eran de mala calidad. Casi tres cuartas partes (280/386) de los participantes convinieron en que era difícil elegir los antibióticos correctos. La mayoría de los participantes (373/386) acogieron bien los programas educativos sobre la prescripción de antibióticos y el 65,0 % (249/383) prefirió pautas locales para el uso de antibióticos frente a las internacionales.

**Conclusión:**

Los médicos en la República Democrática Popular Lao parecen favorecer las intervenciones de prescripción de antibióticos. Las autoridades sanitarias deberían considerar un programa de capacitación que incorpore la prescripción de antibióticos y el control de las infecciones hospitalarias.

## Introduction

Antimicrobial resistance is caused by pathogens changing in ways that render medicines ineffective against the infections they were previously used to treat, and is considered a global public health threat.^1^ Inappropriate use of antibiotics favours selection of resistant bacteria and inadequate infection-control policies facilitate their spread.^1^ One of the factors determining inappropriate use is doctors’ antibiotic prescribing behaviour. Interventions are needed to identify and change these behaviours where needed, rationalize the use of antibiotics and contain further antibiotic resistance. The information to design these interventions may be obtained through knowledge, attitude and practice surveys of prescribers. However, few such surveys have been done specifically on this topic and most were done in the community setting; only two were done in middle-income countries and one in a low-income country.^2^^–^^8^ We report a cross-sectional survey of doctors’ prescribing behaviours in the Lao People's Democratic Republic. Data on antibiotic resistance in the country is scarce, but suggest that resistance is emerging quickly.^9^

## Methods

**Fig. 1:**
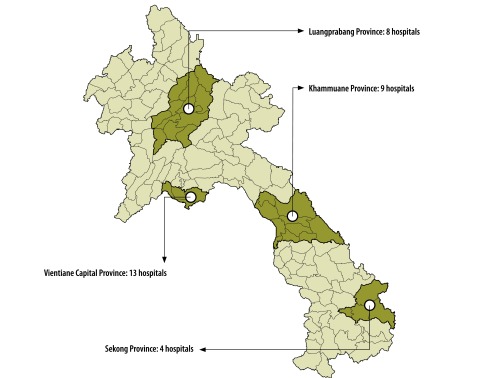
Distribution of study sites in four provinces of Lao People's Democratic Republic, 2012

The health system in the Lao People's Democratic Republic is organized according to the administrative areas in five different referral levels and all hospitals are public. We selected four of 17 provinces – Khammuane, Luangprabang, Sekong and Vientiane capital – that represent the national range of population density, prevalence of poverty (defined as people living below the poverty line, as reported in *The geography of poverty and inequality in the Lao PDR* report)^10^ and service accessibility (Fig. 1 and Table 1). In each selected province, we contacted all hospitals, including the four central tertiary-care teaching hospitals and the medical school in Vientiane capital. However, we excluded the military hospital and the police hospital in the capital.

Image source adapted from the National Geographic Department, Lao People's Democratic Republic.

**Table 1:** Characteristics of the provinces selected for a knowledge, attitude and practice survey, from the Lao People's Democratic Republic DECIDE-Info Project, 2012

Characteristic	Province				Lao People's Democratic Republic
	Vientiane capital	Khammuane	Luangprabang	Sekong	
**Geography**	Centre north, plain	Centre south, plain	North, mountain	South, mountain plateau	_
**Demography**					
Population density, persons per square km	178.1	20.7	24.1	11.1	23.7
% of population living below the poverty line, mean (SD)	29.0 (16.2)	44.2 (15.7)	43.6 (15.8)	60.5 (22.7)	34.7 (NA)
**Service provision**					
No. of hospitals	13	9	9	4	149
No. of medical doctors	311	62	68	22	1250
No. of medical doctors per 100 000 inhabitants	41.3	16.9	15.5	23.2	23.0
Average time to access health service, min (SD)^a^	75.0 (70.8)	66.3 (79.5)	129.6 (108.6)	201.4 (240.2)	45^a^

NA: not applicable; SD: standard deviation.

^a ^74.6% of population reaches health service within 30–60 mins.

Data source: The Lao People's Democratic Republic DECIDE-Info Project (available from: http://www.decide.la/en/applications) – Socio-Economic Atlas of the Lao People's Democratic Republic, an analysis based on the 2005 Population and Housing Census.

Nineteen postgraduate medical doctors participating in the tropical medicine and international health master’s programme at the *Institut de la Francophonie pour la Médecine Tropicale* in Vientiane capital were trained for at least two weeks as surveyors as part of the master’s programme. The training included lectures and practical sessions on addressing public health concerns and survey conduct. No compensation for the training was given, but the surveyors were reimbursed for the costs of travelling to hospitals included in the survey. When training was finished, the surveyors contacted the hospital administrators to arrange an on-site visit during morning meetings between 3 and 12 April 2012. During the meetings, doctors who prescribed antibiotics were invited to fill out the questionnaires anonymously and return them to the surveyors at the end of the meeting. No financial incentive was given to the doctors that participated.

### The survey

The knowledge, attitude and practice survey was based on a recent survey performed in Lima, Peru.^7^ The English version of the questionnaire was reviewed for applicability by a Lao infectious diseases doctor and translated into Lao by a native Lao-speaking medical doctor proficient in English. The translated questionnaire was validated by translating it back to English by a medical doctor who was not involved in the survey preparation.

The questionnaire (available from corresponding author) comprised 43 multiple choice questions related to prescription of antibiotics. They were grouped into seven topics. The first six topics addressed attitudes and practices: (i) demographic and professional profile; (ii) awareness of antibiotic resistance; (iii) sources of information and continuing education on antibiotics; (iv) professional confidence and inputs sought when prescribing antibiotics; (v) factors influencing decisions about antibiotic prescribing; and (vi) the acceptability and appropriateness of potential interventions. For these six topics, answers were presented in a 6-point Likert’s scale, ranging from “strongly agree” to “strongly disagree”, from “very useful” to “not familiar with” or from “always” to “never”. The seventh topic assessed basic knowledge – i.e. spectrum of antibiotics, cross-resistance, pharmacologic properties – as well as case-based questions – i.e. choice, dosage and duration of antibiotic treatment, antibiotic treatment for watery diarrhoea and upper respiratory tract infection, dose adjustments in case of renal failure and safety during pregnancy. Two questions of national relevance – one on melioidosis and one on typhoid fever – were added to the seventh topic. The questionnaire also included two questions assessing knowledge of local antibiotic resistance: the resistance rate of *Salmonella* Typhi to trimethoprim/sulphamethoxazole and the proportion of cases of hospital-acquired pneumonia resistant to cephalosporins.

To prevent repetitive or socially desirable answers, we randomly ordered the first six topics. The knowledge questions were placed in the end of the questionnaire to avoid influencing the answers for the attitude and practice questions.

### Ethical review

The study was reviewed and approved by the Lao Ministry of Health´s ethics committee on 27 February 2012. Written informed consent was deemed not to be necessary, since the study only assessed knowledge, attitude and practice of the participants and data were collected anonymously.

### Statistical analysis

Data were entered by surveyors in a Microsoft Access database created specifically for the study (Microsoft Corporation, Redmond, United States of America). Questionnaires with missing data were included in the analysis.

The six Likert’s items were combined into two categories: “strongly agree/agree”, “very useful/useful” and “always/most of the time” versus the remaining options of the scale unless otherwise stated. Categorical variables were expressed by proportions and their significance was assessed by *χ^2^* tests or Fisher’s exact tests when appropriate. The results of the antibiotic knowledge questions (excluding the two questions about estimations of local antibiotic resistance) were expressed by the values 0 (not correct) or 1 (correct) and a score was calculated for a maximum of 10 points. Continuous variables were expressed by means and standard deviations (SD) and assessed for statistical significance using either Student’s test, the unpaired Wilcoxon’s test or the one-way ANOVA test. Statistical analyses were performed using the software STATA SE 11.1 (StataCorp LP, College station, USA) and 95% confidence interval was calculated. A two-sided *P*-value of ≤ 0.05 was considered as significant.

## Results

The professional and demographic characteristics of participants are presented in Table 2. In total, 25 hospitals were included. Out of the 463 doctors who were asked to fill in the questionnaire, 386 returned it (83.4%). Nearly two-thirds (65.3%; 252/386) of participants had professional experience of five years or more, 22.5% (87/386) and 12.2% (47/386) had experience between two and five years and less than  one year, respectively. Most (99.0%; 382/386) participants recognized that knowledge of antibiotics was important for their profession. Prescriptions of antibiotics at least once a week or at least daily were done by 95.6% (368/385) and 68.3% (263/385) of the doctors, respectively.

**Table 2:** Professional and demographic characteristics of medical doctors participating in a knowledge, attitude and practice survey, Lao People's Democratic Republic, 2012

Characteristic of participants	No. (%)				
	Vientiane capital (*n* = 261)	Khammuane (*n* = 51)	Luangprabang (*n* = 57)	Sekong (*n* = 17)	Total (*n* = 386)
**Hospital**					
Central	222 (85.0)	0	0	0	222 (57.5)
Province	0	24 (47.1)	22 (38.6)	9 (52.9)	55 (14.2)
District A^a^	13 (5.0)	2 (3.9)	30 (52.6)	0	45 (11.6)
District B^b^	26 (10.0)	25 (49.0)	5 (8.8)	8 (47.1)	64 (16.6)
**Professional experience**					
1 year or less	32 (12.3)	7 (13.7)	6 (10.5)	2 (11.8)	47 (12.2)
2 years	34 (13.0)	7 (13.7)	5 (8.8)	2 (11.8)	48 (12.4)
3 years	17 (6.5)	3 (5.9)	0 (0)	1 (5.9)	21 (5.4)
4 years	17 (6.5)	0 (0)	0 (0)	1 (5.9)	18 (4.7)
5 years	20 (7.7)	2 (3.9)	1 (1.8)	1 (5.9)	24 (6.2)
6 years	12 (4.6)	1 (2.0)	2 (3.5)	1 (5.9)	16 (4.2)
≥ 7 years	129 (49.4)	31 (60.8)	43 (75.4)	9 (52.9)	212 (54.9)
**Department**					
Medicine/Emergency	135 (51.7)	37 (72.5)	42 (73.7)	12 (70.5)	226 (58.5)
Surgery	55 (21.1)	3 (5.9)	5 (8.8)	1 (5.9)	64 (16.6)
Paediatric	35 (13.4)	6 (11.8)	2 (3.5)	2 (11.8)	45 (11.7)
Obstetrics/Gynaecology	36 (13.8)	5 (9.8)	8 (14.0)	2 (11.8)	51 (13.2)
**Professional hierarchy**					
Head of service and deputies	41 (15.7)	15 (29.4)	10 (17.5)	8 (47.1)	74 (19.2)
Attending doctor	159 (60.9)	29 (56.9)	38 (66.7)	5 (29.4)	231 (59.8)
Assistant doctor	11 (4.2)	3 (5.9)	9 (15.8)	3 (17.6)	26 (6.7)
Resident	34 (13.1)	2 (3.9)	0 (0.0)	0 (0.0)	36 (9.3)
Intern	16 (6.1)	2 (3.9)	0 (0.0)	1 (5.9)	16 (4.1)
**Acknowledged that knowledge of antibiotics is important in their profession**					
Yes	259 (99.2)	49 (96.1)	57 (100.0)	17 (100.0)	382 (99.0)
No	2 (0.8)	2 (3.9)	0 (0.0)	0 (0.0)	4 (1.0)
**Frequency of antibiotic prescription^c^**					
More than once a day	158 (60.6)	42 (82.4)	35 (62.5)	14 (82.3)	249 (64.7)
Once a day	10 (3.8)	0 (0.0)	4 (7.1)	0 (0.0)	14 (3.6)
3–5 times per week	64 (24.5)	7 (13.7)	10 (17.9)	1 (5.9)	82 (21.3)
1–2 times per week	18 (6.9)	2 (3.9)	2 (3.6)	1 (5.9)	23 (6.0)
Less than once a week	11 (4.2)	0 (0.0)	5 (8.9)	1 (5.9)	17 (4.4)

^a^ Hospitals in district A have technical support for activities of medicine, surgery and obstetrics.

^b^ Hospitals in district B have no technical support.

^c^ In Luangprabang, *n* = 56.

### Antibiotic resistance

Most participants (96.6%; 372/385) agreed that antibiotic resistance is a problem. However, more doctors agreed that the problem was greater globally (323/386) than nationally (288/383) and even fewer doctors saw it as a problem at their hospital (251/385) or in their practice (254/385). Seventy-six percent (293/386) of the participants thought that antibiotics are overused in hospitals as well as in the community setting, however only 47.1% (180/382; *P* = 0.001) agreed so for their own practice. There were significant differences between the four provinces in perception of overuse of antibiotics in the community. Whereas 82.8% (216/261) and 76.5% (13/17) of the doctors in Vientiane capital and Sekong, respectively, thought antibiotics were overused, only 61.4% (35/57) in Luangprabang and 56.9% (29/51) in Khammuane agreed (*P* < 0.001; Table 3).

**Table 3:** The perception and knowledge of antibiotic resistance among doctors, Lao People's Democratic Republic, 2012

Question	Participants agreed/total participants (%)
**Perception of antibiotic resistance**	
Antibiotic resistance is perceived as a problem	
In general	372/385 (96.6)
At the global scale	323/386 (83.7)
At national level	288/383 (75.2)
In their hospital	251/385 (65.2)
In their practice	254/385 (66.0)
Antibiotics are overused	
In hospitals and community	293/386 (75.9)
In their practice	180/382 (47.1)
In Vientiane capital^a^	216/261 (82.8)
In Sekong^a^	13/17 (76.5)
In Luangprabang^a^	35/57 (61.4)
In Khammuane^a^	29/51 (56.9)
In central hospitals^a^	184/222 (82.9)
In provincial hospitals^a^	35/55 (63.6)
In district hospitals^a^	74/109 (67.9)
**Perception of antibiotic quality**	
Agree that some antibiotics are of poor quality in the hospital	148/386 (38.3)
Generic drugs perceived as equivalent to branded drugs	191/378 (50.5)
Generic drugs perceived as substandard drugs	130/378 (34.4)
Generic drugs perceived as counterfeit drugs	21/378 (5.6)
Prescribe antibiotics by international nonproprietary name	213/386 (55.2)
Prescribe antibiotics by brand name	173/386 (44.8)
**Knowledge of antibiotics and their use^a^**	
Methicillin resistant *Staphylococcus aureus* also resistant to cephalosporins	54/386 (14.0)
Metronidazole indicated for anaerobes	270/385 (70.1)
Amoxicillin as a safe antibiotic in the first three weeks of pregnancy	329/384 (85.7)
Ceftriaxone as empiric treatment for bacterial meningitis	303/385 (78.7)
Gentamicin requires dose reduction in patients with renal failure	100/386 (25.9)
Typhoid fever management according to national guidelines	302/386 (78.2)
Melioidosis management according to national guidelines	291/386 (75.4)
Non-febrile diarrhoea should not be treated with antibiotics	315/386 (81.6)
Erroneously reporting that upper respiratory tract infection should be treated with erythromycin	206/386 (53.4)

^a^ Participants only answered for the province/hospital in which they worked.

### Sources of information 

Many participants (59.8%; 231/386) declared not having enough sources of information on antibiotic prescribing and 35.2% (136/386) had not attended any training on antibiotic prescribing during the previous year. Doctors obtained information from national guidelines (86.5%; 332/384), advice from peers (85.1%; 326/383), older colleagues (82.6%; 317/384), pharmaceutical companies (76.9%; 296/385) and the Internet (73.9%; 281/380; Table 4).

**Table 4:** Prescribing practices and potential interventions for doctors in Lao People's Democratic Republic, 2012

Question	Answers/total participants (%)
**Confidence of prescribing**	
Very confident while prescribing antibiotics	190/386 (49.2)
Difficulties with prescribing the correct antibiotics	280/386 (72.5)
**Source of information on antibiotic prescribing**	
Not enough sources of information	231/386 (59.8)
Had not attended training on antibiotic prescribing during the previous year	136/386 (35.2)
Major sources of information	
National Guidelines	332/384 (86.5)
Advice from peers	326/383 (85.1)
Advice from older colleagues	317/384 (82.6)
Pharmaceutical companies	296/385 (76.9)
Internet	281/380 (73.9)
Medical press	16/83 (19.3)
Conferences or seminars	12/83 (14.5)
International antibiotic guidelines	16/83 (19.3)
Advice from specialists	10/83 (12.0)
Information from television	10/83 (12.0)
Use of Internet for prescription advice	
≤ 1 years professional experience	40/45 (88.9)
2 years professional experience	46/48 (95.8)
3 years professional experience	15/21 (71.4)
4 years professional experience	13/18 (72.2)
5 years professional experience	18/24 (75.0)
6 years professional experience	10/14 (71.4)
≥ 7 years professional experience	139/210 (66.2)
In Vientiane capital	215/258 (83.3)
In Khammuane	34/49 (69.4)
In Luangprabang	24/57 (42.1)
In Sekong	8/16 (50.0)
In central hospitals	184/219 (84.0)
In provincial hospitals	38/54 (70.4)
In district hospitals	59/107 (55.1)
**Influences on decision-making**	
Patient demands	195/386 (50.5)
Availability of antibiotics in hospital rather than focus of infection	86/386 (22.3)
Review of prescriptions by a senior colleague	252/386 (65.3)
Restricted drug list stimulating the use of alternative antibiotics	174/385 (45.2)
No list for restricted antibiotics in the hospital	11/385 (2.9)
Inadequate information about antibiotic availability in the hospital	145/385 (37.7)
Unnecessary prescriptions of antibiotics do no harm	115/386 (29.8)
**Potential interventions**	
Prefer local antibiotic guidelines to international guidelines	249/383 (65.0)
Local guidelines are an obstacle	85/386 (22.0)
Restricted use of antibiotics may help to contain antibiotic resistance	174/385 (45.2)
Welcome educational sessions on rational antibiotic use and antibiotic resistance	373/386 (96.6)

Doctors at provincial hospitals cited pharmaceutical companies as an information source more often than doctors in central and district hospitals (92.7%; 51/55 versus, 75.6% 167/221 and 71.5% 78/109 respectively, *P* = 0.021). The use of Internet was more frequently reported by participants with fewer years of professional experience, for example 95.8% (46/48) of the doctors with two years of experience reported using the Internet, while 66.2% (139/210) of the doctors with more than seven years of experience used this source. Furthermore, the Internet was more used in the province of Vientiane capital (83.3%; 215/258) than other provinces (Khammuane: 69.4%; 34/49, Luangprabang: 42.1%; 24/57 and Sekong: 50.0%; 8/16, *P* < 0.001; Table 4).

Half (49.2%; 190/386) of the participants reported that they felt very confident about their prescription of antibiotics, with most of the remaining participants (46.1%; 178/386) replying that they were somewhat confident. However, 72.5% (280/386) agreed that it was difficult to prescribe the correct antibiotics (Table 4).

### Decision-making

About half the participants agreed that patients’ demands contribute to antibiotic prescribing; 49.0% (189/386) and 50.5% (195/386) of participants agreed with this statement for the community and hospital setting, respectively. Eighty-six of the 386 participants (22.3%) declared that antibiotic prescription was more driven by the availability of antibiotics than by the presumed cause of infection and 37.7% (145/385) stated inadequate information about antibiotic availability in their hospital. Review of prescriptions by a senior colleague was reported to occur less than half the time by 65.3% (252/386) of the respondents. Nearly one third (29.8%; 115/386) agreed with the statement that unnecessary prescriptions of antibiotics do no harm, this was more frequent among participants in paediatrics (22/45; 48.9%) as compared to other departments (*P* = 0.013; Table 4).

One hundred and forty-eight of the 386 participants (38.3%) agreed with the statement that some antibiotics available in their hospital are of poor quality (Table 3). Most doctors (55.2%; 213/386) reported prescribing antibiotics by international nonproprietary name, while 44.8% (173/386) prescribed by brand name. Generic antibiotics were perceived as equivalent to branded antibiotics by half of participants (50.5%; 191/378), while 34.4% (130/378) considered such antibiotics as substandard and 5.6% (21/378) as counterfeit products.

### Potential interventions

Local antibiotic guidelines were preferred over international guidelines for 65.0% (249/383) of participants and 22.0% (85/386) of participants considered local antibiotic guidelines as an obstacle rather than a help. One hundred and seventy-four of 385 doctors (45.2%) thought that restriction of antibiotics was an effective measure to contain antibiotic resistance. Most doctors (96.6%; 373/386) welcomed educational programmes on antibiotic prescribing.

### Knowledge

The mean score for the knowledge questionnaires was 5.9 (SD 1.3; range: 2–10). There were no differences in results of the knowledge scores between participants’ provinces, positions in the medical hierarchy, years of experience, frequencies in prescribing, departments or types of referral level.

Table 3 shows knowledge scores. A minority of participants (14.0%; 54/386) knew that methicillin-resistant-*Staphylococcus aureus* (MRSA) was co-resistant to all three generations of cephalosporins. Only 25.9% (100/386) replied correctly to the question about reducing the dose of gentamicin in case of renal failure. The case-based questions about treatment of melioidosis and typhoid fever were correctly answered by 75.4% (291/386) and 78.2% (302/386), respectively. For the question about diarrhoea without fever, 81.6% (315/386) of participants correctly replied that no antibiotic treatment was indicated. In the case of upper respiratory tract infection (rhinitis with sore throat), 53.4% (206/386) reported that they would prescribe erythromycin.

Most doctors had no information about local antibiotic resistance patterns for the pathogens causing typhoid fever and hospital-acquired pneumonia; 54.8% (211/385) and 65.9% (253/384), respectively. Of those who were able to give an estimate of the patterns, the replies were equally distributed among the different proportion categories (0–20%, 30–40% and 40–70%). For both conditions, most participants reported that they based their estimates on clinical experience.

## Discussion

This survey of antibiotic prescribing practices of 386 doctors practicing in four provinces of the Lao People's Democratic Republic, has, like all such surveys, limitations. The multiple-choice format of the questionnaire may have contributed to a social-desirability bias. Not all doctors employed by the hospitals were present at the morning meetings. We focused on hospital doctors and did not assess antibiotic prescribing in the community. Although surveys can provide a snapshot of the situation, additional qualitative research is needed for a more comprehensive understanding of perceptions and practices in antibiotic prescribing.

The strengths of this survey include the balanced selection of provinces, the high coverage of hospitals and doctors from all medical levels, and the high response rate. In addition, the survey included all topics – except for economic incentives and workload issues – cited by the World Health Organization as influencing antibiotic prescribing.^1^ Finally, the questionnaires were distributed and filled in on-site by all doctors attending the morning meeting, precluding access to other sources of information.

### Awareness

As noted elsewhere,^2^^,^^7^ antibiotic resistance was considered a problem by most participants. Even though more doctors thought that the problem was greater at national and global scales than in their own work, more than half answered that antibiotic resistance was an issue in their work. This awareness is noteworthy for a country with very few diagnostic microbiology facilities. Further studies, including quantitative measurements of antibiotic consumption, should be conducted to explore the reasons for the differences in the perception of overuse between the different provinces and between the different types of hospitals.

### Sources of information

As previously recorded in the Democratic Republic of the Congo and Peru,^2^^,^^7^ participants stated that they lacked access to information about prescribing antibiotics. Even though national guidelines were the most frequently-consulted information source, pharmaceutical companies ranked second. This is of concern, since promotional materials in poorly regulated settings may not always be compliant with current evidence-based and ethical standards.^11^ Younger doctors in the capital city were the most likely to use Internet sources, indicating that in the future, distance learning technologies could be a strategy to improve knowledge. 

### Decision-making

Patient pressure was recognized as a factor influencing antibiotic prescribing. However, our study showed this to be less of a factor than reported from the community setting in Peru^7^ while similar to results from the Democratic Republic of the Congo.^2^ The differences may be related to lower economic standards in the Democratic Republic of the Congo and the Lao People's Democratic Republic compared to the urban setting in the Peruvian study. Interventions to educate patients and the general public about antibiotic resistance and the dangers of irrational use are needed to counter the expectations of receiving an antibiotic prescription.^7^

Of particular concern is the high proportion of participants who thought that unnecessary antibiotic prescriptions are harmless for patients; this belief may be driven by fear of treatment failure and lack of laboratory diagnostics.^1^

Two-fifths of the doctors in our study considered that generics were low quality. This perception could be conditioned by reports of sub-standard and counterfeit antimicrobials in the south-east Asian market.^12^^,^^13^ There is on-going debate about the safety and efficacy of generic antibiotics in this context.^14^ Generics have a vital role in standard treatment guidelines and essential medicine lists, provided that they are equivalent to the brand products. Lack of confidence in generics may influence health-care workers and patients to choose more expensive branded products. We recommend that prescription by international nonproprietary name should be encouraged. Quality needs to be assured along the procurement and supply chain and communicated effectively to professionals and the general public.^1^

### Potential interventions

Nearly all participants welcomed educational programmes on antibiotic prescribing and two-thirds of participants preferred local antibiotic guidelines. Since local committees to regulate the prescription and use of antibiotics within hospitals are not functional in the Lao People's Democratic Republic, we did not explore if such committees would be acceptable to doctors. However, antibiotic restrictions were perceived as less efficacious in decreasing the use of antibiotics.

### Knowledge

Participants had higher knowledge scores on the use of antibiotics in life-threatening conditions – such as melioidosis, typhoid fever and meningitis. However, less than half of the participants knew how to correctly manage upper respiratory tract infections. Theoretical knowledge – such as antibiotic spectrum and dose reduction in renal failure – was meagre. Only a minority knew that MRSA is also resistant to cephalosporins. This may partly be explained by the current rarity of MRSA in the Lao People's Democratic Republic.^15^ To our knowledge, diagnostic microbiology is only performed in three hospital laboratories, in the provinces of Khammuane (provincial hospital of Thakhek) and Vientiane capital (Mahosot and Sethathirath hospitals). The small number of microbiological culture facilities could contribute to doctors’ limited knowledge of resistance patterns, not only for MRSA but also *Salmonella* Typhi.

For clear and unbiased surveillance of antibiotic resistance, prospective clinical sampling with well-defined criteria should be implemented nationwide. Such operational studies should be subsidized by the government and integrated with a capacity-building programme that includes antibiotic prescribing and hospital infection control.^16^^–^^19^ The findings from such studies should be disseminated through medical curriculum and continuing medical education and through local medical journals.^9^

## Conclusion

This study identifies action points for improving knowledge and practices of antibiotic prescribers in the Lao People's Democratic Republic. Health authorities should facilitate antibiotic surveillance and provide evidence-based information about local antibiotic resistance and locally-available antibiotics. Introducing a system of quality assurance would reinforce confidence in generic products. Doctors in the Lao People's Democratic Republic appear ready to welcome interventions to improve antibiotic prescribing.
