# Corrigendum

**DOI:** 10.2471/BLT.15.110715

**Published:** 2015-07-01

In Volume 93, Issue 4, April 2015, page 226, the twelfth line in the results section of the French version of the abstract should read: “Près de trois-quarts des participants …”.

